# Calculation of the Free Energy and Cooperativity of Protein Folding

**DOI:** 10.1371/journal.pone.0000446

**Published:** 2007-05-16

**Authors:** Alex Kentsis, Tatyana Gindin, Mihaly Mezei, Roman Osman

**Affiliations:** Department of Molecular Physiology and Biophysics, Mount Sinai School of Medicine, New York University, New York, New York, United States of America; Swiss Federal Institute of Technology Lausanne (EPFL), Switzerland

## Abstract

Calculation of the free energy of protein folding and delineation of its pre-organization are of foremost importance for understanding, predicting and designing biological macromolecules. Here, we introduce an energy smoothing variant of parallel tempering replica exchange Monte Carlo (REMS) that allows for efficient configurational sampling of flexible solutes under the conditions of molecular hydration. Its usage to calculate the thermal stability of a model globular protein, Trp cage TC5b, achieves excellent agreement with experimental measurements. We find that the stability of TC5b is attained through the coupled formation of local and non-local interactions. Remarkably, many of these structures persist at high temperature, concomitant with the origin of native-like configurations and mesostates in an otherwise macroscopically disordered unfolded state. Graph manifold learning reveals that the conversion of these mesostates to the native state is structurally heterogeneous, and that the cooperativity of their formation is encoded largely by the unfolded state ensemble. In all, these studies establish the extent of thermodynamic and structural pre-organization of folding of this model globular protein, and achieve the calculation of macromolecular stability *ab initio*, as required for *ab initio* structure prediction, genome annotation, and drug design.

## Introduction

The importance of accurately defining the molecular ensembles of proteins was recognized early by Levinthal, who concluded that folding of a random coil by way of a diffusive search of its combinatorially vast conformational space is incompatible with the biological energies and timescales of protein folding [1]. Consequently, either the conformational space of unfolded proteins deviates from that of a random coil, or the conformational search is not entirely diffusive, being guided by folding pathway(s), leading to proposals of hierarchical (thermodynamic) and framework (kinetic) folding models, respectively [2]–[5]. The extent to which these two fundamental mechanisms cooperate in the biological attainability of the folding search is unknown.

Structured unfolded states have been observed in a variety proteins [6]–[11]. However, it is unknown whether these macroscopically observed structures correspond to the conformations of individual residues, or to an average of microscopic configurational states that are composed of groups of residues. The former is consistent with the random, albeit conformationally biased (statistical) coil model of the unfolded state, and means that efficient folding is achieved largely by way of kinetic pathways. The latter is not, and implies that the unfolded state is thermodynamically pre-organized. Establishment of the extent of such pre-organization determines the relative contribution of the hierarchical (thermodynamic) and framework (kinetic) folding mechanisms, and is thus of major importance for understanding, predicting and designing biological macromolecules.

Study of this question has been made difficult by the spectroscopic limits of resolving microscopic ensemble sub-states that exist under the conditions of physiologic temperature, pressure, and hydration [12]. Such resolution is achievable theoretically by using molecular mechanics calculations, but is practically limited by the computational limits of simulating proteins in water under physiological conditions. These limits stem precisely from the dependence of conformational sampling of flexible solutes on the molecular properties of the solvent. Conventionally, these limitations are overcome through the implicit treatment of solvent effects, as in the approximation of Born [13]. However, its tested implementations do not appear to reproduce the thermodynamics and structures of natural proteins under physiologic conditions [14], though recently introduced algorithms appear to be more accurate [15]–[17].

Usage of Monte Carlo (MC) algorithms that utilize simultaneous changes of many conformational variables, such as loop torsion MC and replica exchange MC (REM), has shown promise in efficiently calculating convergent ensembles of proteins in aqueous solution [18]–[22]. However, application of loop torsion MC to protein folding depends on the analytical solutions of the loop closure problem, currently available for six polypeptide torsions [23]. REM or parallel tempering MC achieves changes of all conformational variables in aqueous solution through the use of global updates such as molecular dynamics (MD), but requires prohibitively large numbers of replicas in order to generate sufficient energy overlaps, as required by the Metropolis criterion [24], [25]. This Metropolis limit derives from the statistics of energy fluctuations, whereby the energy overlap between adjoining replicas required for efficient MC exchange scales as *n* ^−½^, where *n* is the number of degrees of freedom, which are mostly of bulk water molecules. Recently, partial REM and REM with solute tempering (REST) have been developed to extend the Metropolis limit of REM for the simulations of protein folding in aqueous solution [26], [27]. Both do so by reducing the effective number of the degrees of freedom that contribute to the Metropolis energy criterion.

Here, we introduce another such variant, termed replica exchange MC with energy smoothing (REMS), that does so by manipulating the energy expression itself. We show that in spite of deforming the free energy surface to some extent, REMS yields apparently canonical free energy distributions in the energetic regime of biological systems. Consequently, we apply REMS to simulate the thermal folding of a small globular protein, the 20-residue Trp cage TC5b, under the near physiologic conditions of molecular hydration. We show that such an approach can be used for efficient and accurate calculation of protein stability *ab initio*, at least with respect to the folding of TC5b. And finally, by using self-consistent clustering and machine graph learning, we examine the origin of cooperativity and thermal stability of various structural motifs in this model protein. As a result, we offer a demonstration of the extent of thermodynamic and structural pre-organization of protein folding, important for understanding the mechanics of this phenomenon, with implications for a variety of problems, such as those that require calculations of free energies including structure prediction, genome annotation, and drug design.

## Results and Discussion

### Canonical molecular ensembles in water using REMS

**Figure 1:**
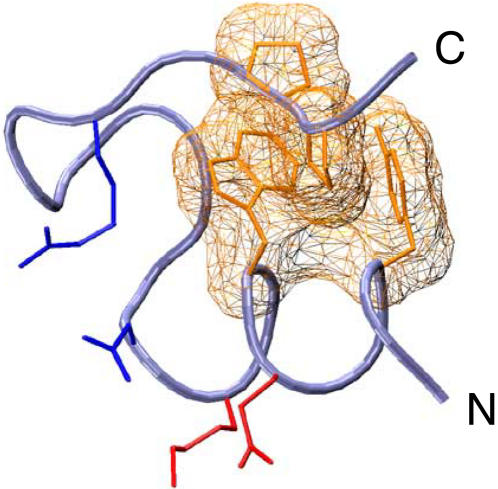
Structure of native TC5b. The structure is composed of an N-terminal α-helix with its α-helical/secondary Q^5^∶K^8^ salt bridge (red), type I β-turn S^13^-S^14^-G^15^ with its β-turn/tertiary D^9^∶R^16^ salt bridge (blue), and a hydrophobic core that includes both α-helical Y^3^∶W^6^ and tertiary W^6^∶P^19^ interactions (gold mesh).

**Figure 2:**
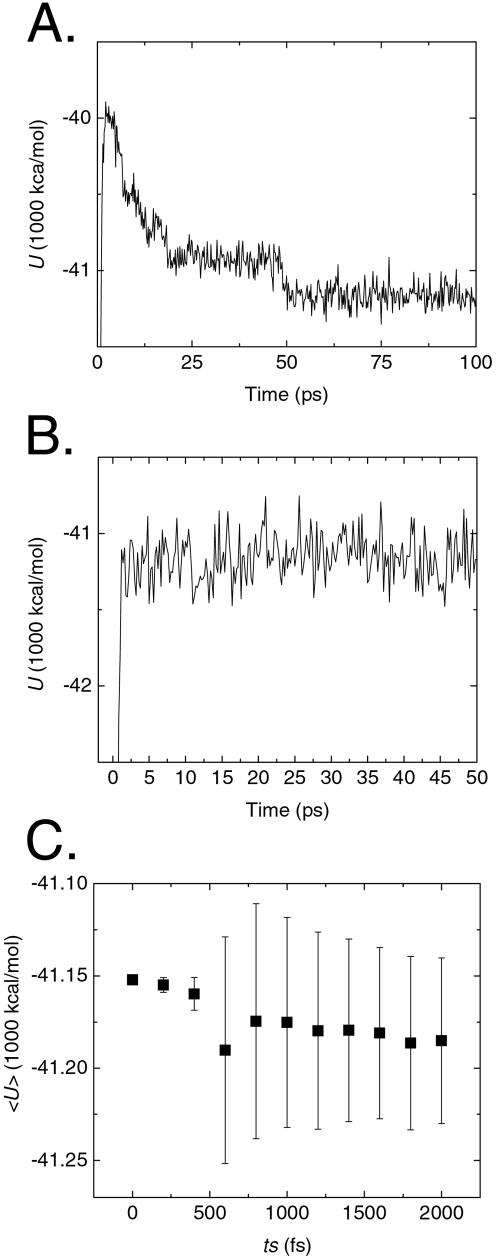
Equilibration and calibration of REMS simulations of TC5b in explicit water. A. Instantaneous potential energy (*U*) as a function of MD time during evolution of the 273 K replica in the canonical *NVT* ensemble prior to initiating REMS, demonstrating its equilibration, as reflected in the energetic stability during the last 50 ps. B. Instantaneous potential energy (*U*) as a function of MD time upon replica exchange from 276 to 273 K, demonstrating thermalization in less than 2 ps. C. Average potential energy 〈*U*〉 of 273 K replica as a function of energy smoothing time (*ts*). As *ts* approaches 2000 fs, the standard deviation of <*U>* approaches the fluctuation of the energy distribution in that time domain. At *ts*  =  200 fs, energy-smoothed 〈*U*〉 of REMS is statistically indistinguishable from the instantaneous *U* used during conventional REM; double-sided *p* = 0.73. Bars represent ±1σ.

TC5b is a small globular protein, consisting of several natural and redesigned structural motifs (Fig. 1). To generate a set of molecular ensembles of the thermal folding of TC5b, we equilibrated 32 replicas of TC5b in explicit water at 273–363 K, corresponding to the temperature range of experimentally measured thermal stability of TC5b [28]. This approach differs from earlier replica exchange simulations of TC5b [29]–[31], in particular by using periodic boundary conditions that are large enough (60×60×60 Å^3^) to accommodate a fully extended TC5b in explicit water, a 100 ps MD trajectory phase prior to replica exchange to achieve equilibration (Fig. 2A), and a 2 ps thermalization time during exchange to prevent quenching (Fig. 2B).

**Figure 3:**
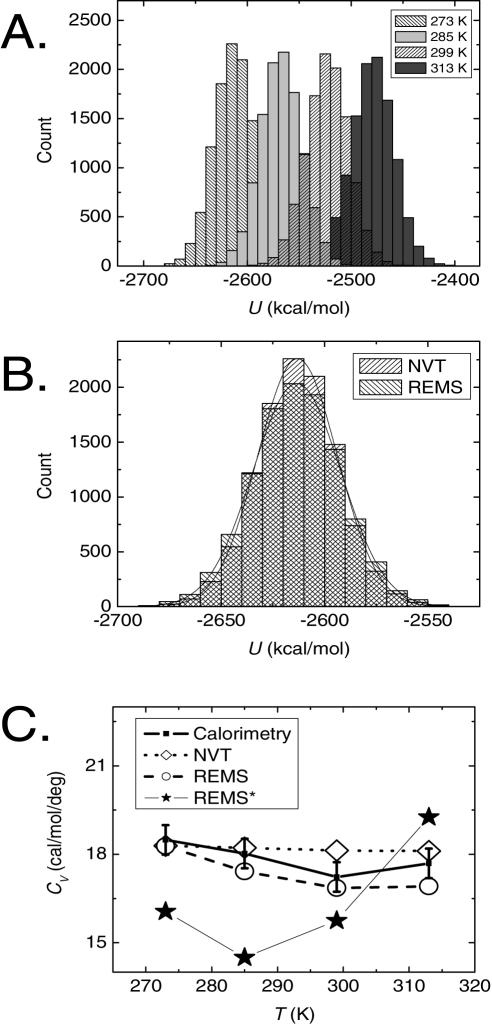
REMS calculation of approximately canonical ensembles of pure water. A. Histograms of potential energies (*U*) of different temperature replicas, demonstrating energy overlaps between adjoining temperature REMS replicas, as required for efficient MC exchange. B. Comparison of histograms of potential energies (*U*) of water ensembles at 273 K calculated using canonical MD (*NVT*) and REMS. Usage of REMS yields statistically indistinguishable mean energies and slightly increased energy fluctuations, as compared to those of canonical MD simulations, as shown by their normal fits (solid curves). C. Heat capacities at constant volume (*C_V_*) of pure water at different temperatures, as obtained experimentally (solid squares), and calculated using canonical MD (dotted diamonds) and REMS (dashed circles). Usage of REMS with extremely long smoothing time of 600 fs (REMS^*^, solid stars) leads to a significant underestimation of the heat capacity of water at low temperature. Sizes of symbols represent ±1σ.

**Figure 4:**
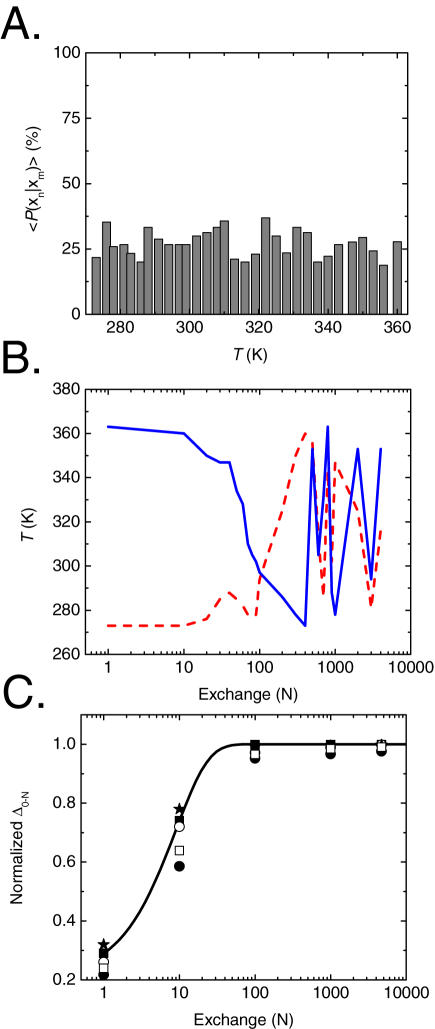
Sampling and efficiency of REMS simulations of TC5b in explicit water. A. Mean probabilities 〈*P*〉 of MC exchange between adjoining replicas *x_n_* and *x_m_* as a function of temperature, demonstrating that usage of REMS leads to efficient replica exchange. B. Exchanges of replicas in the temperature space, tracking the initial lowest (red dashed) and highest (blue solid) containing the predominantly native and unfolded states, respectively, as they diffuse in temperature space in the course of the simulation. C. Divergence of the normalized difference (*Δ*) of fraction of formed hydrophobic core W^6^∶P^19^ (closed squares), hydrophobic core Y^3^∶W^6^ (open circles), salt bridge D^9^∶R^16^ (closed stars), α-helical Y3∶L7 (solid circles) and the β-turn D9∶S14 (open squares) hydrogen bonds between initial and final structures as a function of replica exchange for the 363 K replica. These measure were chosen because their non-local nature should be most sensitive to initial configuration memory effects. The total length of REMS simulation exceeds the apparent computational time constant of self-diffusion by nearly three orders of magnitude.

We used a smoothing time of 200 fs for the calculation of the Metropolis criterion during REMS (Methods), since the smoothed energy at this time shows small fluctuations, and most importantly, preserves approximately Boltzmann-weighted sampling. The difference between smoothed and near instant mean energies is less than 2.8 kcal/mol, and is not significantly different from that at shorter and near instant time intervals (Student's *t*-test *p* = 0.73; Fig. 2C), less than 10% of the total energy of the system. Usage of such energy smoothing leads to a distribution of and a mean potential energy of water (Fig. 3B) as well as temperature dependent heat capacity of water (Fig. 3C) which are statistically indistinguishable from those of exactly canonical simulations. On the other hand, usage of extremely long smoothing time of 600 fs leads to a gross underestimation of water's heat capacity, consistent with significant deviation from canonical sampling (Fig. 3C). Approximately canonical REMS with smoothing time of 200 fs leads to efficient replica exchange with mean exchange probabilities of about 30% (Fig. 4A); conventional REM of this system in explicit water being limited by Metropolis statistics of less than 1% (data not shown). Evolution of the calculated 32 ensembles for more than 4,000 exchanges with mean transition probabilities of about 30% means that the highest and lowest temperature replicas are exchanged on average more than 40 times, as confirmed by tracking the initial lowest and highest temperature replicas, containing the predominantly native and unfolded states, respectively, as they diffuse in temperature space in the course of the simulation (Fig. 4B). Consequently, the final simulation exceeds the apparent computational time constant of self-diffusion of TC5b by nearly three orders of magnitude (Fig. 4C), consistent with the simulation's convergence [21].

### Calculation of thermal stability of TC5b

**Figure 5:**
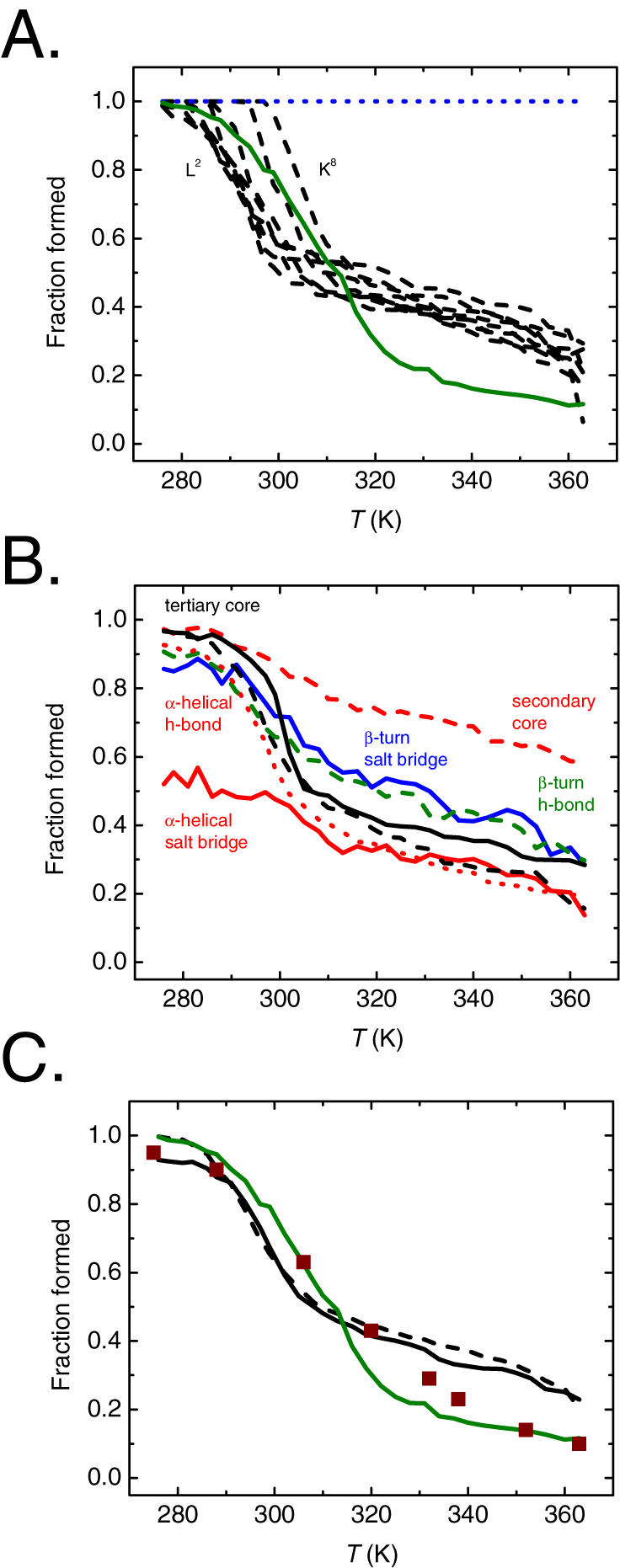
Thermal stability of TC5b. A. Fraction of formed α-helix L^2^YIQWLK^8^ (dashed black), β-turn S^14^ (solid green), and polyprolyl helix P^18^ (dotted blue), as defined using self-consistent clustering and enumeration of their backbone dihedral angles. Note that P^18^ remains unchanged in its backbone conformation due to its definition in CHARMM. Individual α-helical residues have varying thermal stability, with the more N-terminal ones being less stable, consistent with the existence of α-helical fraying. B. Fraction of formed α-helical salt/secondary bridge Q^5^∶K^8^ (solid red), α-helical hydrogen bond Y^3^∶L^7^ (dotted red), β-turn/tertiary salt bridge D^9^∶R^16^ (solid blue), β-turn hydrogen bond D^9^∶S^14^ (dashed green), tertiary hydrophobic core W^6^∶P^19^ and Y^3^∶P^19^ (solid and dashed black), and secondary hydrophobic core Y^3^∶W^6^ (dashed red), as defined by using self-consistent clustering and enumeration of their distances. Note that the α-helical salt/secondary bridge is only partially formed at low temperature, even though the rest of the structure is nearly fully folded by other measures. Similarly, the secondary hydrophobic core Y^3^∶W^6^ persists even at high temperature, where the rest of the protein is largely unfolded by other measures. Importantly, substantial amount of residual native structure persists at high temperature. C. Fraction of formed mean α-helical structure (dashed black), mean β-turn structure (solid green), mean tertiary structure (solid black) in the REMS calculated ensembles, and native fraction measured experimentally using chemical shift dispersion (squares), as adapted from the first study of TC5b [28].

In order to examine the origin of thermal stability of TC5b, we calculated the apparent stabilities of various conformational motifs of TC5b as a function of temperature (Fig. 5). Their choice was guided by the naturally occurring secondary and tertiary structural elements, as well as those that were specifically redesigned in TC5b [28] (Fig. 1). At 273 K, REMS calculated conformational ensemble of TC5b is largely folded, with nearly all molecules forming the N-terminal α-helix, the β-turn, the C-terminal polyprolyl helix (Fig. 5A), and tertiary and secondary hydrophobic cores (Fig. 5B). In contrast to the average NMR structure, the β-turn hydrogen bond and salt bridge, as well as the α-helical hydrogen bonds are largely (∼90%) but not persistently formed. On the other hand, the α-helical salt bridge is formed only in half of the ensemble (Fig. 5B), in agreement with the experimental observations of TC5b [28], [32]–[34].

Stabilities of both local and non-local structural motifs exhibit an apparently sigmoid melting transition (Fig. 5). In particular, the N-terminal α-helix melts with an apparent melting temperature of 300–310 K, depending on the exact residue monitored, consistent with the presence of N-terminal fraying, wherein the helical residues closer to the terminus are less stable (Fig. 5A). Similarly, the β-turn melts with an apparent melting temperature of about 310 K (Fig. 5A), associated with the destabilization of the tertiary core and the β-turn/tertiary salt bridge (Fig. 5B). Calculation of the overall melting temperature of TC5b, by using mean ensemble conformational statistics, where all conformational motifs are equally weighted, yields an apparent value of approximately 310 K (Fig. 5C), in good agreement with the experimentally measured value of 315 K [28]. This finding differs from those of earlier replica exchange simulations of TC5b, which overestimated the apparent melting temperature by about 100 K, possibly because of continuum Born solvation [30], or constricted boundary conditions [29]. However, it is important to note that our study is limited by the use of a single (CHARMM) force field and initial (native) conditions, which may bias and limit sampling, respectively.

### Unfolded state ensemble

Experimental studies of TC5b indicate a substantial amount of residual structure in the unfolded state ensemble at high temperature [28]. Our calculated high temperature ensemble also exhibits such structures, in particular possessing up to about 10% β-turn, 20% α-helical content, and 30% tertiary hydrophobic core (Fig. 5). Remarkably, nearly 60% of molecules in the high temperature ensemble contain the secondary hydrophobic core (Fig. 5B), in agreement with the experimental findings of such residual structure, as observed by using both NMR and fluorescence spectroscopies [28], [32]. This residual structure may arise from the persistence of various native-like conformations (isolated α-helical turn or hydrophobic core) in different molecules of the unfolded state ensemble that is otherwise non-native and heterogeneous. Conversely, this residual structure may be due to configurations of groups of conformations (associated α-helix and hydrophobic core) in an unfolded state ensemble that is relatively homogeneous with respect to these native-like configurations. Though indistinguishable macroscopically, these characteristically composed molecular ensembles diverge in the ways they affect protein folding and stability.

### Graph manifold learning of the unfolded state ensemble

**Figure 6:**
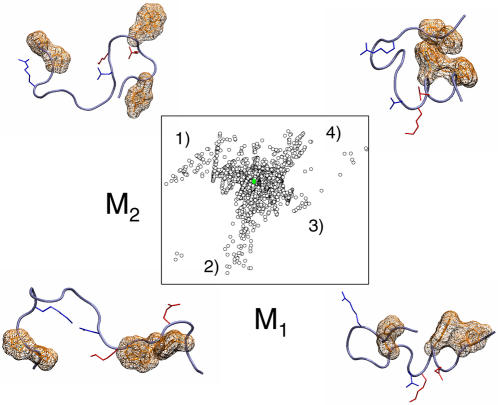
Manifold of unfolded mesostates. Mapping of the unfolded state ensemble, as calculated using the 363 K replica, onto the two top coordinates of its locally linear embedding space (open black circles), and the two top coordinates of its principal component projection (solid green circles). Principle component analysis fails to discern mesostate structure of the unfolded state ensemble, with the entire ensemble located near the origin of the PCA projection. On the other hand, displacement along the manifold from the origin of the LLE map coincides with the formation of native-like mesostates, containing: 1) α-helical/secondary salt bridge (red), 2) β-turn/tertiary salt bridge (blue), 3) α-helix and α-helical hydrophobic core, and 4) nearly native configurations with both the α-helix and the tertiary hydrophobic core.

In order to discover the origin of residual structure at high temperature, we applied a graph-based approach designed to learn the natural coordinates of highly dimensioned data. By embedding the molecular ensemble in a graph based on geometric similarity, and projecting the individual structures onto a manifold that preserves nearest-neighbor geometric relations of this graph, we are able to distinguish globally organized configurations, termed mesostates, from groups of structures comprised of unrelated conformations (Methods). Indeed, the high temperature manifold is comprised of several such mesostates, including configurations of secondary structures such as the N-terminal α-helix and the β-turn, as well as more complex configurations that contain both the α-helix and the tertiary hydrophobic core, for example (Fig. 6). These configurations are not due to the use of the NMR structure as the starting configuration for REMS, as the latter's memory is lost after about 30 replica exchanges and the final ensemble is evolved for more than 4,000 exchanges (Fig. 4B&C). Instead, these configurations appear to pre-organize the unfolded state ensemble for folding by virtue of arranging individual interactions and conformations in the context of native-like mesostates. This pre-organization is likely inherent to the polypeptide sequence of TC5b, as suggested by energy minimization calculations of fragments of TC5b [35].

### Folding cooperativity and pre-organization

In order to estimate the extent of pre-organization of the thermal folding of TC5b by the residual structure of the high temperature ensemble, we calculated the apparent cooperativities of forming pairs of conformations into configurations, as expressed by the probabilities of forming these configurations conditional on the formation of their constituent conformations (Table 1). The conditional probabilities of forming these four-body interactions are related to the mesostate organization of the thermal folding reaction. Consistent with the presence of configurations containing the N-terminal α-helix in the high temperature ensemble (Fig. 6), the apparent conditional probability of forming both the α-helical hydrogen bond and the α-helical salt bridge exceeds the expected probability of forming this configuration assuming independence of its constituent conformations by more than a factor of 10 (Table 1). Similar effect is observed for the apparent cooperativities of formation of other configurations, involving both the secondary and tertiary hydrophobic cores and β-turn (Table 1). This surprising phenomenon is likely due to the thermodynamic coupling between the formation of individual native-like conformations and their organized configurations, such that native-like conformations are adopted essentially in the context of topologically native configurations.

**Table 1:** Folding cooperativity in the unfolded state ensemble of TC5b

	***P_pair_***	***P_i_***	***P_j_***	***P_pair_*** **/** ***P_i_ P_j_***
**α bridge+α hbond**	0.30	0.14	0.19	11
**α hbond+α core**	0.54	0.19	0.59	4.8
**α core+3° core**	0.27	0.59	0.16	2.9
**3° core+β hbond**	0.29	0.16	0.27	6.7
**β hbond+3° bridge**	0.51	0.27	0.29	6.5

Conditional probabilities of forming pairs of native interactions, as listed, with *P_pair_* = *P* (*i*+*j*|*i* ; *j*), the probability of forming both interactions *i* and *j* under the condition that either *i* or *j* is formed. The overall probabilities of forming individual interactions *i* and *j* are defined by *P_i_* and *P_j_*, respectively, and the product *P_i_* *P_j_* expresses the probability of forming both interactions *i* and *j* in the absence of any cooperativity between them. This cooperativity is expressed by the ratio *P_pair_*/*P_i_* *P_j_*.

Apparent coupling between local (conformational) and non-local (configurational) contacts has been noted earlier during the folding of Gō lattice polymers, where its origins were related to the details of the potential energy function defining the native state [36]–[38]. As the Gō protein model is supplemented with backbone interactions, local backbone conformations can lead to progressive non-local organization [39]. The finding of analogous conformational-configurational coupling during the folding of TC5b (Table 1), where on the other hand the folding process is defined by an atomic polypeptide in the context of a semi-empirical, classical force field (Methods), suggests that such coupling is inherent to the properties of the hydrated polypeptide itself.

The apparent cooperativity between forming concomitant α-helical and tertiary hydrophobic cores of TC5b exceeds the expected non-cooperative value by nearly a factor of 3 (Table 1). Because the formation of both α-helical and tertiary hydrophobic cores defines most of the native topology of TC5b (Fig. 1), this suggests that the residual unfolded state structure in the form of native-like configurations and mesostates at high temperature (Fig. 6) is responsible for most of the folding search. This phenomenon is similar to the pre-organization of α-helix formation in hydrated polyalanine [40], for which the microscopically pre-organized unfolded state contributes as much as half to the folding search [21], [41]. Altogether, the findings of such extensive pre-organization of both secondary structures as well as globular proteins suggest that the apparent biological efficiency of protein folding is due in large part to the thermodynamic pre-organization, as opposed to kinetic guidance. This pre-organization acts to reduce the conformational space available to the diffusive search of the unfolded state ensembles that are pre-ordered in configurational mesostates.

### Graph manifold learning of folding mesostates

**Figure 7:**
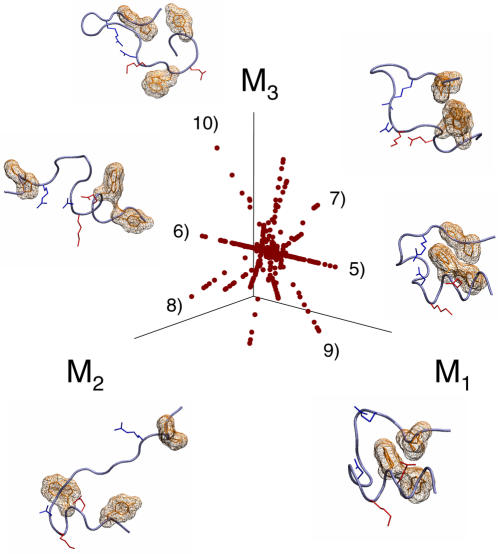
Folding reaction manifold. Mapping of TC5b's folding ensemble at the midpoint of its thermal transition, as calculated using the 310 K replica, onto the top three coordinates of its LLE manifold. Displacement along the *M_1_* coordinate of the manifold coincides with the transformation of the 5) nearly native and 6) partially unfolded mesostates that lack the tertiary hydrophobic core and the native β-turn, but retain a frayed α-helix and the tertiary salt bridge. Displacement along the *M_2_* coordinate coincides in part with the transformation of the α-helix from mesostate 7) that possesses a near native β-turn and hydrophobic cores and a non-α-helical but compact N-terminus, and mesostate 8) that lacks the native hydrophobic cores and has a non-native β-turn centered at K^8^ that is part of the N-terminal α-helix in the NMR structure. Displacement along the *M_3_* coordinate coincides with the transformation of the β-turn, including mesostates 9) that have a near native β-turn and tertiary salt bridge but have an unfolded α-helix and hydrophobic cores, and 10) possess a near native topology and α-helix but lack a native β-turn.

In order to assess how the thermal folding reaction can proceed by way of configurational mesostates, we examined the folding ensemble at the midpoint of its folding transition as comprised by the 310 K replica, by using graph manifold learning. At the folding midpoint, the unfolded and native state ensembles are equi-populated, and their inter-conversion defines all of the possible folding pathways [42]. Projections of individual configurations of the folding ensemble onto its LLE space map a star-shaped manifold, with multiple mesostates radiating from the origin of the projection (Fig. 7). Displacement along the *M_1_* coordinate of the manifold coincides with the transformation between the native and unfolded structures, with configurations near the origin of the LLE map being partially native-like (Fig. 7). Displacements along the *M_2_* and *M_3_* coordinates coincide in part with the transformations of the α-helix and the β-turn, respectively, either in the context of native-like or unfolded topologies, depending on the particular location along the *M_1_* coordinate (Fig. 7). The LLE mapping identifies a wide variety of folding mesostates, including those that possess a near native topology and α-helix but lack a native β-turn, those that lack the tertiary hydrophobic core and the native β-turn but retain the α-helix, as well as those that possess a near native β-turn and hydrophobic cores but lack the α-helix (Fig. 7). The existence of such mesostates explains the observed stabilities of their constituent conformational motifs (Fig. 5), as well as the apparent cooperativities of their configurations (Table 1). Combined with the star-shaped organization of the manifold of TC5b's folding ensemble, the variety of these folding mesostates suggest that the thermal folding reaction of TC5b is structurally heterogeneous. Though the folding of TC5b is pre-organized extensively by the unfolded state ensemble (Table 1&Fig. 6), this heterogeneity implies a relative diversity of available folding pathways, in agreement with experimental studies [43]. Determination of the exact subset(s) of folding mesostates that contribute to the kinetic transition state ensemble and the overall folding mechanism is an important direction of future work.

### Conclusions

Insofar as the free energy of flexible polymers can be described by a configurational partition function, our study shows that molecularly adapted variants of replica exchange, including REMS introduced here, can be used for the calculation of the free energy and cooperativity of protein folding *ab initio*. In addition, structural configurations and mesostates unknown *a priori* but adopted by the folding ensemble can be discovered and characterized by using graph manifold learning methods such as LLE. Our findings indicate that the thermal folding of a model globular protein, Trp cage TC5b, involves a structurally heterogeneous set of configurations and mesostates (Fig. 7). Some of these configurations persist in the molecular ensemble at high temperature (Fig. 6), concomitant with the pre-organization of TC5b's folding by such ordered unfolded state ensemble (Table 1). Combined with observations of thermodynamic pre-organization of polypeptide secondary structures [21], [41], these findings suggest that macromolecular modularity, as described by ensemble mesostates, likely plays an essential role in determining the structures and stabilities of biological macromolecules.

Furthermore, the successes and failures of current *de novo* protein design approaches likely reflect the significance of configurational organization of protein ensembles and the latter's contribution to protein stability, respectively [44]. Since TC5b's thermal stability and apparent folding cooperativity, two hallmark features of equilibrium folding of all natural proteins, are related to the residual structure of TC5b's unfolded state ensemble (Figs. 5&6), this suggests that the design of naturally stable proteins may be based on the structural preferences of unfolded polypeptides [45], as obtained computationally for example [46]. Indeed, TC5b has been re-designed recently by using just such an approach [47]. Application of advanced methods such as replica exchange Monte Carlo to sample the rugged energy spaces of proteins, and graph manifold learning to analyze the vast structural spaces of the molecular ensembles that constitute them, should prove useful for a variety of *ab initio* approaches to structure prediction, genome annotation, and drug design.

## Methods

### Molecular systems

To understand the origin of protein stability and cooperativity, we chose to examine a protein the folding of which is well characterized structurally, thermodynamically, and kinetically. The smallest such protein is the 20-residue Trp cage [28], TC5b (NLYIQWLKDGGPSSGRPPPS; Fig. 1), a derivative of the Gila monster extendin-4 that has been truncated and redesigned to include an N-terminal α-helix cap (N^1^), α-helical/secondary salt bridge (Q^5^∶K^8^), β-turn/tertiary salt bridge (D^9^∶R^16^), and optimized hydrophobic stack (Y^3^∶W^6^). In addition, TC5b contains a naturally occurring type I β-turn S^13^-S^14^-G^15^, type II polyproline helix P^17^-P^18^-P^19^, and a hydrophobic core containing both local secondary L^2^-Y^3^-I^4^ and non-local tertiary W^6^∶P^18^ and Y^3^∶P^19^ interactions.

NMR structure of TC5b (PDB code 1L2Y; model 1) was used as the starting configuration for our studies. The structure was solvated under periodic boundary conditions using a 60×60×60 Å^3^ cubic box of equilibrated TIP3 water, and energy minimized using the CHARMM27 potential energy function in the presence of one randomly placed chloride ion to yield electroneutrality [48], [49]. The resulting system was heated using molecular dynamics with a linear gradient of 20 K/ps and equilibrated in the isothermal-isobaric (*NPT*) ensemble at 273 K and 1 atm pressure for 100 ps, using the Leapfrog Verlet integrator with velocity rescaling, 2 fs integration time step, energy-conserving Nose-Hoover thermostat, SHAKE to constrain hydrogen atoms, center of mass updates to remove rotational and translational solute motion, and particle mesh Ewald (PME) summation to calculate electrostatic interactions, as implemented in CHARMM. Because these calculations were already in progress, we did not use the subsequently introduced CHARMM backbone dihedral parameter correction [50]. Upon equilibration, systems were 60.7×60.7×60.7 Å^3^ in volume, containing a total of 21,640 atoms and 7,112 water molecules. Such size and equilibration was necessary to thermalize and unfold this protein (see below). This system was used as the initial state for molecular dynamics equilibrations in the canonical (*NVT*) ensemble for 100 ps at mean temperatures of 273+3*n* K, where *n* = (0, 31).

### Replica exchange

For REM, we utilized the MMTSB Tool Set, a recently developed collection of Perl scripts that interface with CHARMM [51]. Thirty two replicas were prepared as described above, thermalized at temperatures that were spaced between 273 and 363 K, a range chosen based on the experimentally observed thermostability of TC5b [28]. Each replica was simulated independently in the canonical ensemble under periodic boundary conditions using Leapfrog Verlet molecular dynamics with velocity rescaling, 2 fs integration time step, Nose-Hoover thermostat, SHAKE constraint, and PME electrostatics. Every 2 ps, an exchange between replicas *n* and *m* neighboring in temperature was attempted using the energy smoothed Metropolis criterion: *P* (*x_n_* | *x_m_*) = 1 if Δ*E* ≤ 0 and *P* (*x_n_* | *x_m_*) = exp(–Δ*E*) if Δ*E*>0, where *P* is the probability of exchange, Δ*E* = *β_n_* 〈*U_n_*〉*_ts_*–*β_m_* 〈*U_m_*〉*_ts_*, *β* = 1/k_B_ *T*, *U* is potential energy, and *ts* is the MD smoothing time preceding the exchange over which the energies of the instantaneous configurations *x_n_* and *x_m_* are box averaged. Value of *ts* was tuned interactively to produce mean exchange acceptance rates of about 30%, while preserving approximately Bolzmann sampling, with *ts* of 200 fs used to generate the results described below. In the limit where the smoothing time is very long and the distribution of energy approaches the fluctuations in that time domain, e.g., *ts*>600 fs (Fig. 2C), REMS is expected to produce ensembles with significantly reduced energy differences among states, allowing transitions between states that would otherwise by very different in their energies.

Upon each exchange of replicas neighboring in temperature, another exchange using the new pairs of neighboring replicas was attempted in order to maximize the tempering effect and the movement of replicas across the sampled temperature range. Upon a completed exchange, velocities of the exchanged configurations were rescaled to the new temperatures, another exchange was attempted 2 ps later, and the entire REMS simulation was produced for a total of 4,710 exchanges, while discarding 100 initial exchanges, corresponding to more than 0.3 µs of aggregate MD time, and sampling more than 150 million configurations.

Energy smoothing of REMS is equivalent to introducing an error into the calculation of the Metropolis criterion, and consequently produces non-stationary distributions of Markov chains of configurations. Though different in origin, this feature of REMS is analogous to the lack of stationary distributions produced by other tempering methods such as variants of Jump-walking (J-walking), where the conventional MC walker is allowed large transitions sampled from a different temperature ensemble, yielding generally non-stationary distributions of states [52], [53]. Nevertheless, it can be shown that approximately canonical distributions of states can be generated by using tempering MC methods such as J-walking when the jumping frequency is low compared to the total length of the MC walker, e.g., when the deformation of the free energy of the system is small relative to the differences in energy of major ensemble states [54], [55].

In order to evaluate the suitability of REMS to actually recover canonical energy distributions, we calculated the constant volume heat capacity of pure water: C_v_ = (〈U^2^〉–〈U〉^2^)/k_B_T^2^. Because heat capacity reports squares of energy fluctuations, it is an extremely sensitive measure of the equipartition of energy that characterizes canonical ensembles. For this purpose, we used a 20×20×20 Å^3^ box of equilibrated TIP3 water under periodic boundary conditions, simulated using MD in the canonical ensemble for 1 ns, using MD protocol as described above, at four different temperatures: 273, 285, 299, and 313 K. We carried out a REMS simulation of the same system, using replicas at 273, 285, 299, and 313 K, simulated for 1,000 exchanges attempted every 1 ps with *ts* of 200 fs, corresponding to aggregate MD time of 1 ns, equal to that of simulations using canonical MD without REMS. As can be seen from Fig. 3, usage of the energy smoothed Metropolis criterion does not lead to any significant distortions of the mean energy of water under near physiologic conditions of temperature and pressure, as seen from the comparison of the results of MD NVT and REMS simulations. More importantly, no statistically significant differences exist between the constant volume heat capacities of water calculated using exactly canonical MD NVT and approximately canonical REMS, both of which are in good agreement with the experimentally measured values (Fig. 3) [48].

To evaluate the computational efficiency of REMS, we calculated mean transition probabilities of exchanging pairs of replicas adjoining in temperature during the course of the simulation of the thermal folding of TC5b. As can be seen from Fig. 4, usage of REMS improves the otherwise system size-limited parallel tempering MC, yielding mean exchange acceptance ratios of about 30%, similar to traditional MC transition probabilities. To evaluate sampling efficiency, we calculated the evolution of the apparent self-diffusion coefficient *Δ_0−N_* = (*f*(0) − *f*(*N*))/*f*(final), as a function of simulation length with respect to the number of replica exchanges *N*, where *f* is a phase space variable, such as the fraction of the native hydrophobic core of TC5b. If the sampling of phase space is ergodic, *Δ_0−N_* decays to one at long *N*. This is a necessary but insufficient condition of ergodicity, since it depends on the choice of initial and final conditions. Due to the requirement of carrying out multiple independent simulations, we are unable to evaluate ergodicity directly [21].

### Microscopic analysis and clustering

In the analysis of structures of calculated ensembles, we use the term conformation to refer to geometries of individual interactions, and configuration to refer to molecular geometries of groups of interactions. Although canonical structures, such as α-helices and β-turns, have defined regular geometries, conformations in solution at ambient temperature exhibit considerable plasticity. Thus, we utilized a self-consistent method for defining conformational basins using a stepwise optimal clustering algorithm based on a self-organizing neural net, as implemented in ART-2 by Brooks and coworkers [56]. Briefly, the cluster assignments of structural variables extracted from simulation ensembles were minimized subject to a constraint on a cluster radius, such that no member of a cluster was farther than a specified distance from the cluster center. Because the convergence of such minimizations is sensitive to initial conditions, we tested the robustness of assignments to conformational basins by recalculating cluster assignments using reshuffled trajectories (data not shown). Cluster occurrences and probabilities of sampling of conformational basins as defined in this manner were calculated using a set of home-built programs, available upon request.

In this manner, we examined the formation of the N-terminal α-helix by clustering (*Φ,ψ*) dihedral angles of the L^2^YIQWLK^9^ polypeptide backbone and intrahelical hydrogen bond distances between backbone amide hydrogens and carbonyl oxygens, formation of the α-helical/secondary Q^5^∶K^8^ salt bridge by clustering the distance between side chain Q carboxamide oxygen and K amine nitrogen, formation of the β-turn by clustering (*Φ,ψ*) dihedral angles of S^14^ and the hydrogen bond distance between backbone D^9^ carbonyl oxygen and side chain S^14^ hydroxyl hydrogen, formation of the β-turn/tertiary salt bridge D^9^∶R^16^ by clustering the distance between side chain D carboxylate carbon and R guanidino nitrogen, formation of the polyproline helix by clustering (*Φ,ψ,ω*) dihedral angles of P^18^, and lastly, formation of the hydrophobic core by clustering contact distances among side chain Y^3^ phenol carbon ζ, W^6^ indole carbon δ, and P^19^ imido carbon δ. For all conformational variables, probabilities of forming native conformations were calculated by using clusters with near native centroids, as referenced to the NMR structure of TC5b.

### Folding manifold learning

Because probabilities of forming structural configurations, such as folding intermediates, cannot be derived from conformational probabilities *a priori*, we examined their occurrence by direct enumeration of conditional probabilities of forming pairs of conformations. Apparent cooperativities of forming pairs of native interactions were calculated by using *P_pair_* = *P* (*i*+*j*|*i* ; *j*), the probability of forming both interactions *i* and *j* under the condition that either *i* or *j* is formed.

In order to discover configurations that involve more than four-body interactions described above, we applied non-linear graph manifold learning techniques. Conventionally, study of high dimensional data such as atomic protein folding trajectories has been done using linear methods such as principal component analysis (PCA). PCA works by computing linear projections of greatest variance from the top eigenvectors of the data covariance matrix, thereby preserving the covariance structure of the data. However, because the global structure of high dimensional data is not necessarily linear, low dimensional linear principal components fail to capture this structure adequately (Fig. 6) [57]. Recently, graph based methods, including locally linear embedding (LLE), have been developed to preserve data neighbor relationships without enforcing global linearity [58]. Simply put, such methods provide compact representations of complex data without imposing artificial constraints.

Our LLE input data set was dimensioned using the Cartesian coordinates of heavy atoms of TC5b (154 atoms × 3 (*x,y,z*) = 462 dimensions), and included 2,355 configurations sampled from the 363 K replica to model the unfolded state ensemble, or from the 310 K replica to model the folding ensemble. All coordinates were centered and oriented with respect to the NMR structure of TC5b (PDB model 1, see Methods) in order to simplify the calculated manifolds, though this procedure is not required, in contrast to PCA [59], [60]. LLE was carried out by calculating Euclidean distances between individual configurations, as defined by the Cartesian coordinates of their heavy atoms, and constructing nearest neighbor graphs using *k*-means clustering to define nodes of *k*-nearest neighbors. For the results shown below, we used *k* of 18. Varying *k* between 12 and 20 produced no qualitative differences in resulting manifolds (data not shown). The constructed graphs contained edges that specified nearest neighbor relations, as based on geometric similarity of Cartesian coordinates of heavy atoms, with edge weights computed by reconstructing each input configuration *x_i_* from its *k*-nearest neighbors and minimizing the reconstruction error *ε_W_* = Σ*_i_* (*x_i_*−Σ*_j_* *W_ij_x_j_*)^2^. The low dimensional manifold that preserved these locally linear neighbor relations was constructed by minimizing *ε_ψ_* = Σ*_i_* (*ψ_i_*−Σ*_j_* *W_ij_ψ_j_*)^2^, where *ψ_i_* is the low dimensional embedding of the high dimensional configuration *x_i_*. Such a manifold preserves distance relationships of the data, subject to the constraints of the nearest-neighbor graph and the locally (but not globally) linear embeddings that describe it.

Our approach is related to other graph-based studies of molecular ensembles [61]–[63], but instead of analyzing kinetic or energetic relations among states with respect to each other, we examine their geometric (dis)similarities with respect to the overall organization of the ensemble. In this way, projections of the high dimensional configurations *x_i_*, as sampled from their molecular ensembles, onto the low dimensional manifold *ψ_i_* reveal groups of geometric mesostates and their ensemble coordinates.
